# Detection of Carbohydrate Antigen 50 Based on a Novel Miniaturized Chemiluminescence Analyzer Enables Large-Scale Cancer Early Screening in Grassroots Community

**DOI:** 10.3389/fbioe.2022.920972

**Published:** 2022-07-07

**Authors:** Yu Liu, Xiaowei He, Jingjing Zou, Xiuyun Ouyang, Chunrong Huang, Xiao Yang, Yu Wang

**Affiliations:** ^1^ South China University of Technology, Guangzhou, China; ^2^ National & Local United Engineering Lab of Rapid Diagnostic Test, Guangzhou Wondfo Biotech Co., Ltd., Guangzhou, China; ^3^ Department of Laboratory Medicine, Guangzhou First People’s Hospital, South China University of Technology, Guangzhou, China

**Keywords:** miniaturized analyzer, point-of-care detection, chemiluminescence immunoassay, carbohydrate antigen 50, large-scale screening of cancer

## Abstract

Early screening of cancer can effectively prolong survival time and reduce cancer mortality. However, the existing health-monitoring devices can only be carried out in professional laboratories, so large-scale early cancer screening in resource-limited settings is hardly achieved. To embrace the challenge, we developed a novel chemiluminescence immunoassay (CLIA) analyzer that does not require a professional operation. Then, it was applied to detect carbohydrate antigen 50 (CA50), a non–organ-specific tumor marker for screening various cancers. As a result, the analyzer exhibited excellent performance that the total assay time was only 15 min, and the detection limit reached 0.057 U ml^−1^. A coefficient of variance (*CV*) less than 15% was well-controlled for both intra- and inter-assay precision, and the linear range was 0–500 U ml^−1^. More importantly, this analyzer can continuously detect 60 samples per hour without any professional paramedic. Finally, this analyzer has been applied to evaluate clinical samples and the detected results showed a good correlation with the clinical test results (correlation coefficient, 0.9958). These characteristics exactly meet large-scale and high-throughput early screening of cancer. Thus, this miniaturized analyzer for CA50 detection is promising to achieve early large-scale screening of cancer in the resource-limited grassroots community.

## 1 Introduction

Cancer is a leading cause of death in every country of the world (Bray et al., 2021). It is estimated that 19.3 million new cancer cases and almost 10.0 million cancer deaths occurred worldwide, including lung cancer (18%), colorectal cancer (9.4%), liver cancer (8.3%), stomach cancer (7.7%), and female breast cancer (6.9%), according to the “Global cancer statistics” (Sung et al., 2021). Encouragingly, the “Cancer Statistics, 2021” pointed out that the cancer death rate in America has fallen continuously from its peak in 1991 through 2018, for a total decline of 31%, because of the improvements in early detection and treatment (Siegel et al., 2021). Multiple randomized controlled trials have demonstrated that early screening can effectively prolong the survival time and reduce cancer mortality (de Koning et al., 2020; Duffy et al., 2020). Promoting the early screening technology for cancer provides timely intervention and treatment for more cancer patients, which can significantly dismantle the existing health disparities and give everyone the opportunity to be as healthy as possible. However, the shortage of medical resources and skilled professionals becomes the main obstacle to popularize early screening of cancer. Most cancer patients cannot be found at cancer early-stage in resource-limited settings, such as the grassroots community and countryside. Therefore, to enable comprehensive diagnostic applications and routine surveillance, it is urgent to promote detection technologies that are rapid and of low cost for the large-scale screening of cancer.

To achieve this vision, there are critical challenges in several aspects: 1) throughput and turnaround time, a large number of samples in large-scale screening of cancer requires the high-throughput system to detect multiple samples, simultaneously. Short turnaround time is critical to ensure rapid screening, which can win precious time for the diagnosis and treatment of patients (Liu et al., 2019; Huang et al., 2021). 2) Portable and low-cost analysis instrument, a miniaturized and simplified point-of-care (POC) analyzer, occupying a small area and being easy to move and carry, and can provide a flexible detection service without the limitation of time and space (Qu et al., 2020; Morioka et al., 2021; Huang et al., 2022). 3) Automated operating system, grassroots communities lack professional medical staff; therefore, the automated operating system can reduce manual error and decrease the probability of misdiagnosis (Li et al., 2021).

Chemiluminescence immunoassay (CLIA) is an assay that combines the chemiluminescence technique with immunochemical reactions and is widely used in clinical diagnosis because of its high sensitivity, rapidity, cost-effective, and easy automatization. (Ishibashi et al., 2019; Li et al., 2020; Qi et al., 2020). However, a conventional fully automated CLIA analyzer is routinely performed in professional clinical laboratories equipped with huge instruments and paramedics (Cetin et al., 2014; Yan et al., 2019), which limits the application of CLIA in large-scale screening of cancer in resource-limited settings, such as the MAGLUMI^®^800 CLIA analyzer (Luang et al., 2020) and the Abbott i2000 CLIA analyzer (Kim et al., 2007). Although some CLIA methods have also been coupled with the other technologies (e.g., microfluidic chips or quantum dots) (Xiao and Xu, 2020) to overcome this bottleneck, such as a microarray CLIA for the detection of IgG antibodies to SARS-CoV-2 (Klüpfel et al., 2021), a microfluidic paper-based device for early diagnosis of acute myocardial infarction (Yang et al., 2018), and a droplet-array microfluidics-based CLIA for POC detection of procalcitonin (Huang et al., 2022), an apparent drawback was the requirement for professionals to pretreat the samples and manually add immunoreagents due to the lack of an intelligent and integrated operating system (Dai et al., 2021; Klüpfel et al., 2021; Li et al., 2021; Huang et al., 2022). Large-scale screening in the grassroots communities requires not only miniature and portable analyzers but also intelligent and integrated operating systems. Hence, the miniaturized CLIA analyzer with a fully automated operation system and shortened analysis time has been urgently demanded for the rapid POC cancer screening (Poschenrieder et al., 2019; Xiao and Xu, 2020; Liu et al., 2021).

In this work, we described a novel fully automated miniaturized CLIA analyzer, which is mainly aimed at a large-scale health screening in grassroots communities. This analyzer was automatically disposed and collected samples and detected without professional operators. Just by loading the samples in the platform and applying for detection items, the results would be obtained in 15 min, and the detection throughput also reached 60 T h^−1^. We systematically evaluated its clinical detection performance using CA50, a non–organ-specific tumor marker, which showed a high correlation with many types of cancers (Shan et al., 2019; Luang et al., 2020). Inspiringly, the sensitivity of the CA50 detection reached 0.057 U ml^−1^ and maintained stability and accuracy during testing. Moreover, we evaluated the novel miniaturized analyzer with clinical serum samples, and then, the correlation coefficient with the clinical test results was 0.9958. Taken together, the novel miniaturized analyzer can promote the fully automated CA50 detection to enable early large-scale screening of cancer in the grassroots community.

## 2 Materials and Methods

### 2.1 Apparatus and Materials

The fully automated CLIA miniaturized analyzer was developed by Wondfo Biotech Co., Ltd. (Guangzhou, China). The magnetic separator was provided by Thermo Fisher Scientific Inc. (Shanghai, China). Sephadex G-25 was bought from GE Healthcare Life Sciences (Beijing, China). Constant incubation and shaking procedures at 37°C were conducted at a temperature and humidity incubator (HWM-358, Jiangnan instrument Co., LTD, Ningbo, China). Ultra-pure water obtained from a Milli-Q water purification system (Millipore, Bedford, MA, United States), was used throughout the study. Anti-CA50 monoclonal antibodies (A1 & A2, B1 & B2, C1 & C2, and D1 & D2) were provided by Wondfo Biotech Co., Ltd. (Guangzhou, China). Carbohydrate antigen 50 (CA50), carbohydrate antigen 199 (CA199), carbohydrate antigen 125 (CA125), and carbohydrate antigen 242 (CA242) were purchased from Fapon Biotech Co., Ltd. (Shengzhen, China). Alkaline phosphatase (ALP) was procured from BBI Co., Ltd. (China). The substrate was provided by Wondfo Biotech Co., Ltd (Guangzhou, China). The magnetic particles (MPs) suspended in solution and 1-(3-dimethylaminopropyl)-3-ethyl carbodiimide hydrochloride (EDC) were purchased from Merck (Beijing, China). N-Succinimidyl 4-(N-maleimidomethyl) cyclohexane-1-carboxylate (SMCC) used as the cross-linking agent and dithiothreitol (DTT) used as the reductant were obtained from Thermo Fisher Scientific Inc., (Shanghai, China). The clinical serum samples determined by the commercial CLIA analyzer (MAGLUMI^®^800) from SNIBE company were provided by the clinical laboratory of Wondfo Biotech Co., Ltd.

### 2.2 Establishment of CLIA for CA50

First, the optimal anti-CA50 antibody pair was selected as the coating antibody and labeling antibody, respectively. (Supplementary Figure S1). Second, the immunoconjugate of MP-coated anti-CA50 antibodies (MPs-Ab) was prepared according to the previously reported method (Ren et al., 2015) with a little modification. ALP-labeled CA50 antibodies (ALP-Ab) were prepared, as described previously (Du Ye and Peng, 2015), with minor modifications. The detailed steps for immunoconjugate preparation are shown in Section 1.1 of the Supplementary Material. The core parameters of the coating technology including the MP type, activator amount, concentration of coating antibodies on MPs, and coupling time were systematically optimized by evaluating the RLU and coupling efficiency (Supplementary Figure S2), and the cross-linker type and concentration involved in the labeling technology for the preparation of ALP-Ab were also optimized by comparing the RLU. (Supplementary Figure S3). Additionally, the stability of immunoconjugates would significantly affect the sensitivity and accuracy of the immunoassay. Therefore, the stability of MPs-Ab and ALP-Ab was further evaluated through the accelerated stability experiment (Supplementary Table S1). The details are also displayed in the Supplementary Material.

### 2.3 Fully Automated Miniaturized Analyzer

**FIGURE 1:**
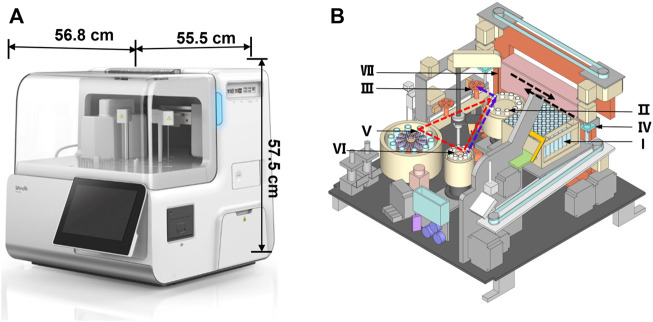
Fully automated miniaturized analyzer **(A)** and the front view of the interior structure **(B)**. The number Ι -Ⅶ represented the tube storage device (Ι), rotational incubation device (Ⅱ), magnetic separation device (Ⅲ), grab device (Ⅳ), reagent storage device (Ⅴ), sample storage device (Ⅵ), and sample/reagent collection device (Ⅶ), respectively. The black dotted arrow represented the direction of the device Ⅳ; the red dotted triangle represented the layout of the devicesⅡ, Ⅴ, and Ⅵ; the purple dotted arrow represented the movement track of the sample.

The miniaturized analyzer has a volume of only 0.18 m^3^ and weighs 60 kg (Figure 1A), which is portable and convenient for large-scale health screening in the grassroots community. It is mainly made up of a sample processing module, reagent processing module, reaction processing module, and consumable processing module, which cooperate to realize the fully automated operation. The interior structure diagram schematic of the miniaturized analyzer is shown in Figure 1B, which was repeatedly optimized and tested to ensure high performance. The analyzer possessed the following distinctive features to meet the requirements of POC detection. First, the tube storage device (Ⅰ), rotational incubation (Ⅱ), magnetic separation (Ⅲ), and the interior optical detection device are located side by side so that the grasp device (Ⅳ) could move linearly among the abovementioned devices in the direction shown by the black dotted arrow in Figure 1B. The design of linear movement of the grasp device not only saved the interior space of the analyzer but also shortened the run time spent on moving back and forth among the devices Ⅰ-Ⅲ. Second, a highly integrated and miniaturized interior structure was designed to further reduce the size and weight of the analyzer. Briefly, the main core devices including the reagent storage (Ⅴ), sample storage (Ⅵ), and the rotational incubation devices (Ⅱ) formed a distinctive triangular layout shown by the red dotted triangle in Figure 1B, contributing to a more compact layout and then dramatically reducing the footprint of the overall equipment. Third, the rotational incubation (Ⅱ), reagent storage (Ⅴ), and sample storage (Ⅵ) devices were, respectively, set at 10 positions to support the detection of 10 items and 10 samples simultaneously, providing high-throughput POC detection for multiple biomarkers. Notably, the fully automated and intelligent operation system without any manual operation greatly shortens the total analysis time.

The specific analytical process of the analyzer includes 1) antigen capture: the grab device (Ⅳ) transfers the tube from the storage device (Ⅰ) to the incubation device (Ⅱ); then the collection device (Ⅶ) pipettes successively the sample and reaction reagents into the tube. After shaking, the reaction tubes are incubated at 37°C controlled by the inner temperature control device; 2) magnetic separation: the grab device (Ⅳ) transfers the tube containing the sandwich model of immune complexes to the magnetic separation device (Ⅲ), washing extra reagents by a magnetic bar capturing magnetic beads; 3) luminescence reaction: the tube is transferred to the optical acquisition device, and the substrate containing AMPPD is added into the tube, where the light signal occurs soon; and 4) signal detection: the light signal acquired is converted into an electrical signal and then amplified through the photomultiplier tube inside the instrument, and the signal is detected by the detector automatically. The fully automated operation system removes the complicated operating procedures, reducing the total assay time significantly. The design features of the novel analyzer exactly meet the requirements of rapid POC detection.

### 2.4 Immunoassay and Operation Procedures of the Miniaturized Analyzer

**FIGURE 2:**
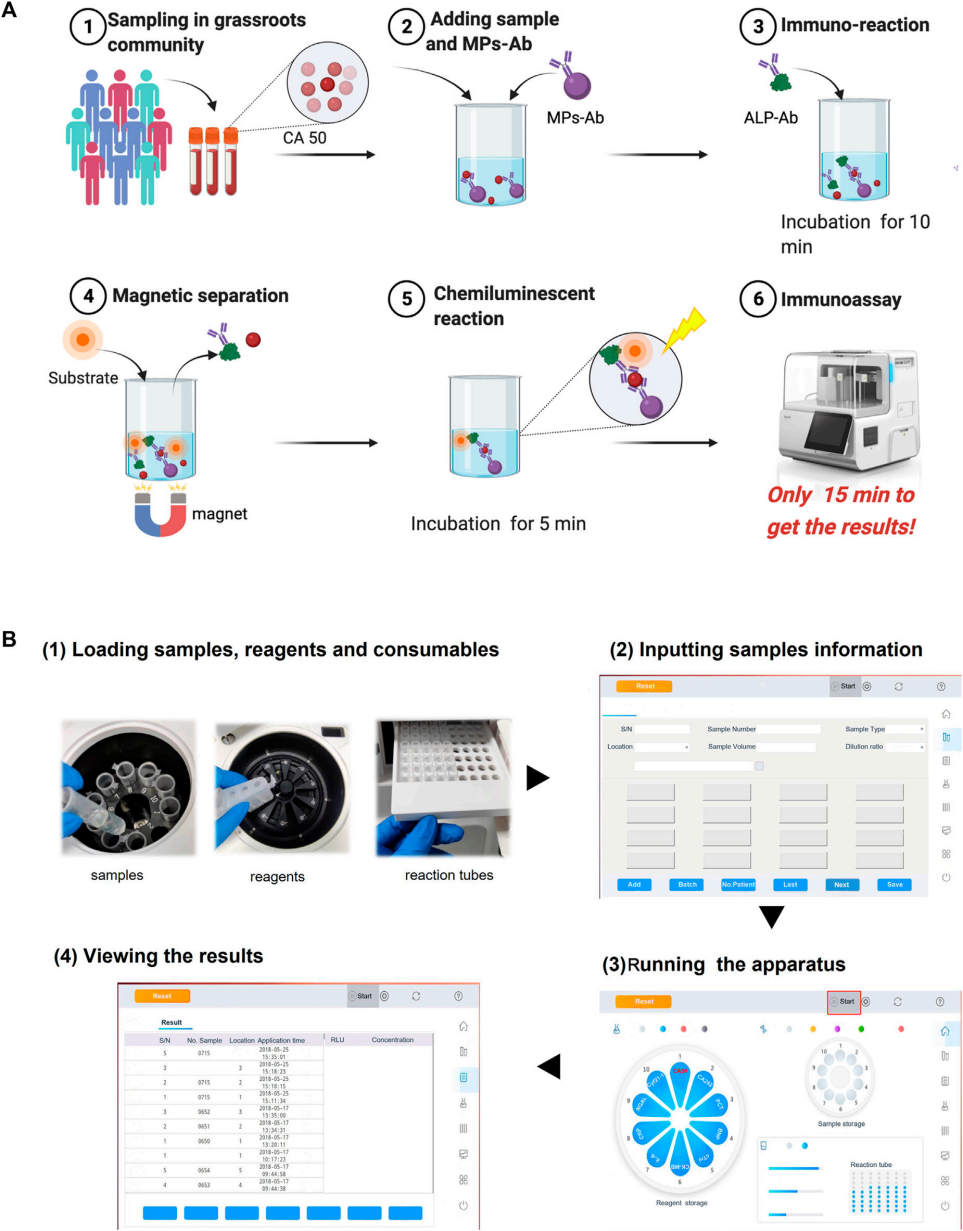
Schematic illustration of the proposed POC-CLIA for CA50 detection **(A)** and the operation procedure of the miniaturized analyzer for users **(B)**.

The method proposed in this work was based on the novel miniaturized analyzer aiming to realize the POC chemiluminescence immunoassay (POC-CLIA) using a one-step sandwich method. The detailed schematic reaction protocol is shown in Figure 2A. After large-scale sampling, the serum samples were assayed according to the following steps. Briefly, 10 μl serum samples, 50 μl MPs-Ab (dilution ratio of 1:50), and 50 μl ALP-Ab (dilution ratio of 1:500) were in turn added to the reaction tube. Then, the mixture was incubated with shaking at 37°C for 10 min. After that, the sandwich model of immune complexes (MPs-Ab/CA50/Ab-ALP) was formed, which was attracted by the magnets. Afterward, the mixture was washed to remove excess ALP-Ab and CA50. Then, 200 μl of the luminescent substrate containing AMPPD was introduced and incubated with the sandwich compounds for 5 min at 37°C. Finally, the emitted photons were measured as the relative luminescence unit (RLU).

There are just four simple steps of the entire analytical process for the user using the miniaturized analyzer from sample-in to result-out. As shown in Figure 2B, it included 1) loading reagents, samples, and reactions tubes; 2) inputting sample information; 3) running the apparatus; and 4) viewing the results. Finally, the user can get the results in 15 min.

### 2.5 Clinical Application and Concordance Test

A total of 50 negative samples and 64 positive samples, provided by the clinical laboratory of Guangzhou Wondfo Biotech Co., Ltd., were performed in coincidence rate analysis and the Kappa consistency test on 114 samples. The calculation is as follows:

$$\text{Positive}\;\text{coincidence}\;\text{rate}\left(\text{\%}\right)=\frac{\text{a}}{\text{a}+\text{c}}\times 100\text{\%}$$ (1)

$$\text{Negtive}\;\text{coincidence}\;\text{rate}\left(\text{\%}\right)=\frac{\text{d}}{\text{b}+\text{d}}\times 100\text{\%}$$ (2)

$$P_{0}=\frac{\text{a}+\text{d}}{\text{a}+\text{b}+\text{c}+\text{d}}\times 100\text{\%}$$ (3)

$$P_{e}=\frac{\left(\text{a}+\text{b}\right)\left(\text{a}+\text{c}\right)+\left(\text{c}+\text{d}\right)\left(\text{b}+\text{d}\right)}{{\text{n}}^{2}}$$ (4)

$$\text{Kappa}=\frac{P_{0}-P_{e}}{1-P_{e}}$$ (5)

where a and d represent the coincident positive and negative samples, respectively; c and b are the numbers of false-positive and negative samples, respectively; n is the sum of a, b, c, and d; *P*_*0*_ is the actual coincidence rate, and *P*_*e*_ is the expected coincidence rate. The acceptable range of coincidence rate should be >90%, and the Kappa value should be 0.6–1.0. When the value exceeds 0.8, it means excellent concordance (Kwiecien et al., 2011).

### 2.6 Statistical Analysis

All data are presented as the mean ± standard deviation (SD) from three replicates. The calibration curves were obtained by linearly fitting the CA50 concentration and the RLU or dilution rate by Origin 9.0 software (OriginLab, Massachusetts, United States), and the correlation between the detected concentration and the actual concentration of clinical sample was also analyzed by linear fitting by Origin 9.0 software. Statistical analyses were carried out by SPSS Statistics V22 software (IBM, New York, United States). Statistical significance among the experimental data was performed using an LSD *t*-test or one-way ANOVA within *p* < 0.05.

## 3 Results and Discussion

### 3.1 Optimization of Immunoassay Conditions

We performed rapid POC detection of CA50 using the miniaturized CLIA analyzer for large-scale cancer screening. To improve the performance of POC-CLIA, the concentration and volume of the immunoreagents, the volume of the sample, and incubation time were optimized using the positive (S7, 200 U ml^−1^) and negative samples (S0, 0 U ml^−1^). The RLU and the ratio of RLU_S7_/RLU_S0_ served as the parameter for the determination of the best assay.

#### 3.1.1 Optimization of the MPs-Ab and ALP-Ab Concentrations

**FIGURE 3:**
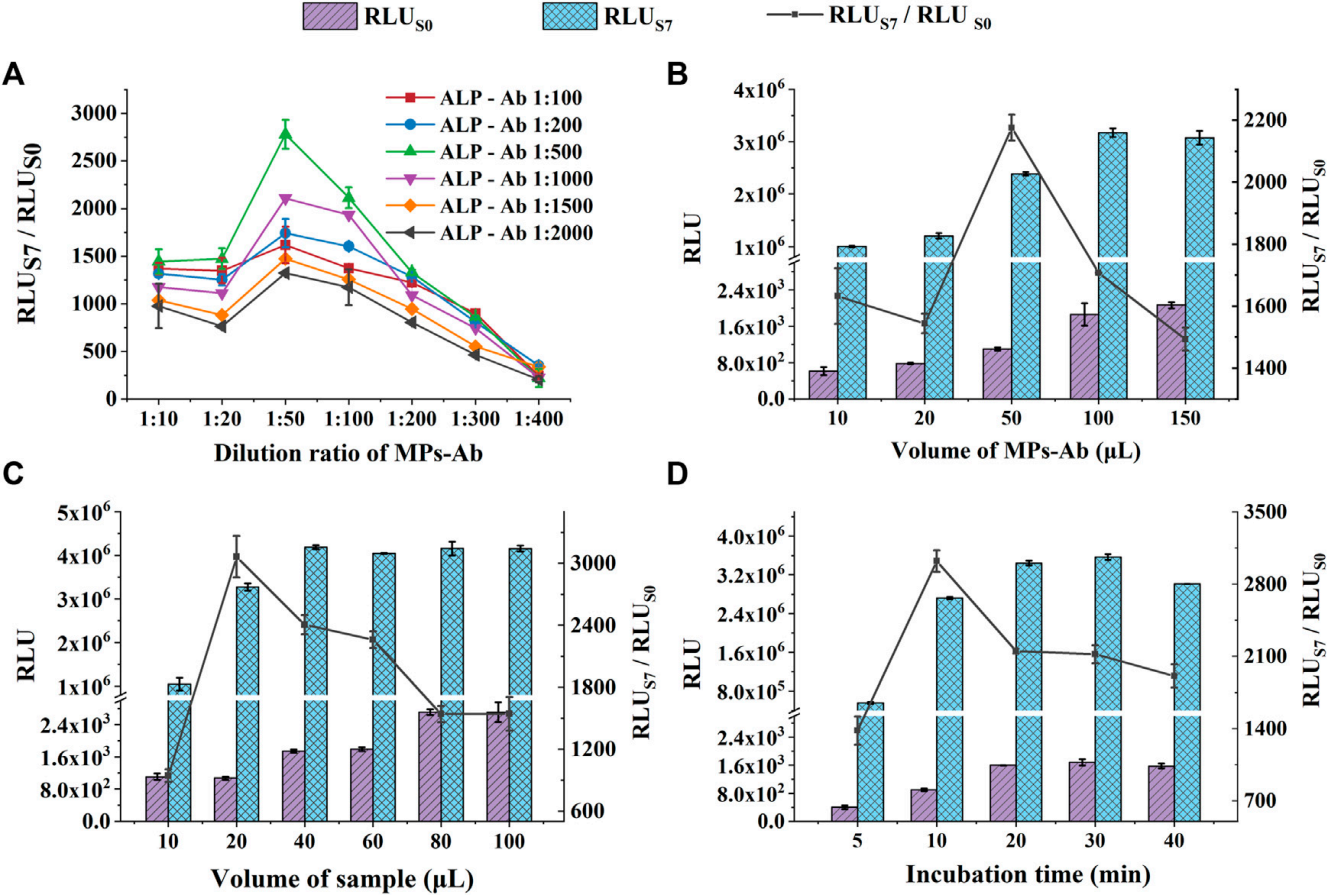
Optimization of experimental conditions for CA50 detection: the dilution ratio of MPs-Ab and ALP-Ab **(A)**; the volume of MPs-Ab **(B)**; the volume of the sample **(C)**; and incubation time **(D)**. The concentrations of S0 and S7 used in these experiments were all 0 U ml^−1^ and 200 U ml^−1^, respectively.

The concentration of immunoreagents would affect the sensitivity and accuracy of the immunoassay, especially in a sandwich immunoassay (Wang et al., 2019). Therefore, we optimized the dilution ratios of MPs-Ab and ALP-Ab. In Figure 3A, with a gradient dilution of MPs-Ab, the RLU_S7_/RLU_S0_ increased first (1:10–1:50), reached a peak (1:50), and then decreased (1:50–1:400). These results indicated that MPs-Ab of 1:50 added was sufficient for binding the antigen to MPs-Ab, resulting in the highest RLU_S7_/RLU_S0_. Increasing the concentration of MPs-Ab above 1:50 led to lower sensitivity caused by the blocking of luminescence and non-specific adsorption due to excess MPs-Ab (Wang et al., 2007; Liu et al., 2013). Additionally, RLU_S7_/RLU_S0_ increased with the dilution ratio of ALP-Ab from 1:100 to 1:500 and decreased with the ratio from 1:1,000 to 1:2,000. These results might be attributed to ALP-Ab with a low concentration, which did not bind sufficient CA50, while a high concentration of ALP-Ab would result in high background and poor sensitivity. Therefore, we selected the dilution ratios of 1:50 and 1:500 for MPs-Ab and ALP-Ab, respectively, to further prepare the detection system for CA50.

#### 3.1.2 Optimization of the Volume of MPs-Ab

The effect of the volume of MPs-Ab was studied, and the results are shown in Figure 3B. RLU_S0_ and RLU_S7_ increased significantly with the MPs-Ab volume up to 50 μl (*p* < 0.05). However, RLU_S7_ kept stable, but RLU_S0_ increased continuously with the volume from 50 to 150 μl. This might be attributed to the excessive amount of MPs, which leads to higher RLU of the blank sample (lower sensitivity) because the color of MPs was black and the monolayer of MPs formed in the tube bottom could absorb the emitted light unavoidably (Liu et al., 2013; Shan et al., 2017). Additionally, the increased RLU_S0_ also resulted in a significantly decreased ratio of RLU_S7_/RLU_S0_ under the MPs volume above 50 μl (*p* < 0.05). It was also observed that the ratio of RLU_S7_/RLU_S0_ reached a peak at the volume of 50 μl, but showed a decrease, inconsistent with the increased RLU, under the volume from 10 to 20 μl. This might be attributed to the insufficient amount of MPs-Ab that could not capture enough antigens, resulting in the unsaturated luminescence intensity of RLU_S7_ (Shan et al., 2017). Therefore, to obtain both a high value of RLU_S7_/RLU_S0_ and sensitivity, the following experiments were executed using MPs-Ab with a volume of 50 μl.

#### 3.1.3 Optimization of the Volume of Sample

The effect of the sample volume on CLIA performance was investigated. We set up six groups with various volumes from 5 to 100 μl. As presented in Figure 3C, the RLUs of S0 and S7 both increased with the increasing sample volume; then, RLU_S7_ reached the peak at the volume of 40 μl, suggesting that a high amount of sample improves the antigen-antibody binding efficiency. However, the high volume caused a low sensitivity due to the increasing RLU_S0_. In comparison, the small volume of 20 μl produced weaker luminescence intensity, but the highest ratio of RLU_S7_/RLU_S0_, indicating a relatively low volume of the sample, was beneficial for detecting sensitivity and accuracy. The previous study also reported that the low amount of sample led to the upper sensitivity of procalcitonin (Huang et al., 2022). Additionally, the low sample consumption was exactly satisfied with the requirement of the large-scale POC screening of cancer. Therefore, samples with a volume of 20 μl were chosen in subsequent experiments.

#### 3.1.4 Optimization of Incubation Time

A suitable incubation time for the antigen-antibody is beneficial to improve the overall reaction efficiency and save the assay time that is extremely critical for the large-scale screening of cancer (Nayak et al., 2017; Liu et al., 2021). As shown in Figure 3D, the RLU increased within 20 min and hardly changed in 20–30 min, indicating a binding equilibrium between the antigen and antibody. After that, a longer incubation time of 40 min resulted in a considerably reduced RLU, which might be due to a dissociation of the sandwich immune complex. Although the RLU reached its peak in 20 min incubation time, the highest RLU_S7_/RLU_S0_ of 3,023 was achieved in 10 min (*p* < 0.05). The results suggested that RLU_S7_ and RLU_S0_ at 10 min was enough to distinguish a positive sample at the cut-off value from the negative control. More importantly, the short assay time is the most distinctive feature of POC detection (Liu et al., 2019). Therefore, the incubation time of 10 min was selected, considering both the suitable RLU and the requirements of rapid POC screening. Under the optimized experimental conditions, the entire assay process, including incubation for the immunoreaction (10 min) and luminescence reaction (5 min), can be completed within 15 min. The rapid analysis can be owed to the fully automated operation system of the miniaturized CLIA analyzer.

### 3.2 Performance of the Miniaturized CLIA Analyzer for CA50 Detection

Performance of POC testing of CA50 based on the miniaturized CLIA analyzer was evaluated by determining the limit of detection (LOD), precision, cross-reactivity, recovery, linearity, and hook effect.

#### 3.2.1 Standard Calibration Curve

**FIGURE 4:**
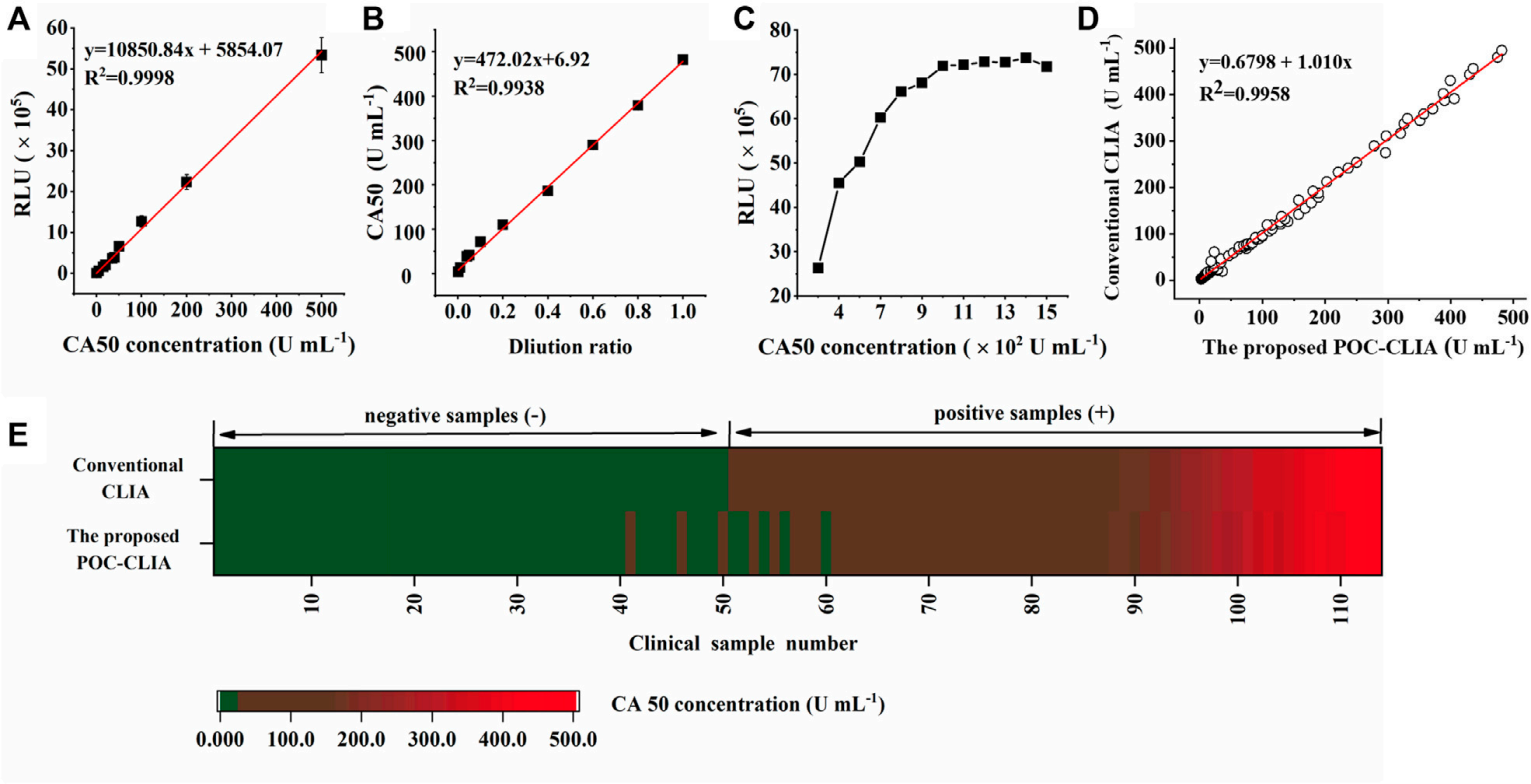
Evaluation of the performance of the miniaturized CLIA analyzer for CA50 detection: standard calibration curve **(A)**; linearity-dilution effect **(B)**; hook effect **(C)**; and clinical performance **(D,E)**. The color green represents the negative sample containing CA50 below 25 U ml^−1^, and red means the positive sample containing CA50 over 25 U ml^−1^, respectively.

Under optimal conditions, a series of CA50 standard samples (0, 5, 15, 20, 35, 40, 50, 100, 200, and 500 U ml^−1^) were detected by the miniaturized CLIA analyzer. The standard calibration curve (y = 10,850.84x + 5,854.07) is shown in Figure 4A, which was obtained by linearly fitting the CA50 concentrations (x) and RLU (y). The coefficient (*R* ^2^) was determined to be 0.9998, indicating a good correlation between the CA50 concentration and RLU within the range from 0 to 500 U ml^−1^. The limit of detection (LOD) is calculated to be 0.057 U ml^−1^, which was obtained by testing five blank samples (S0) for four consecutive days (20 replicates) and then calculating the average concentration (S0) with double the corresponding SD for 20 replicates (Feng et al., 2014). These features of our newly developed analyzer are within the required parameters for use in the clinical diagnostic of CA50.

#### 3.2.2 Precision

Precision has often been emphasized in quantitative analysis. The precision is closely related to the accuracy and repeatability of immunoassay. To evaluate the precision, three CA50 standard samples were analyzed using the miniaturized CLIA analyzer. The intra-assay precision was calculated by analyzing samples with the concentrations of 2.00, 20.00, and 200.00 U ml^−1^ five times per run in 1 day. Similarly, the samples were analyzed 25 times on different days to obtain the interassay precision (Liu et al., 2013). Table 1 showed that the *CVs* of interassay and intra-assay were all below 10%, indicating the high reproducibility of the proposed POC detection of CA50.

**TABLE 1:** Intra-assay and inter-assay tests of the fully automated miniaturized analyzer.

Times	Theoretical concentration (U ml^−1^)	CA50 concentration (U ml^−1^)		
		AV (U ml^−1^)	SD	*CV %*
Intra-assay (n = 5)	2.00	2.46	0.05	2.03
	20.00	25.98	0.94	3.62
	200.00	221.72	3.18	1.43
Inter-assay (n = 25)	2.00	2.58	0.23	8.91
	20.00	24.89	1.07	4.30
	200.00	222.58	9.77	4.39

#### 3.2.3 Cross-Reactivity and Accuracy

To evaluate the specificity for CA50 detection with the miniaturized CLIA analyzer, some possible interferences in the serum samples were tested, such as triglyceride (15 mg ml^−1^), human hemoglobin (22 mg ml^−1^), and bilirubin (0.66 mg ml^−1^). The cross-reactivity (CR) was 1.67%–3.85% (Table 3). In addition, various related tumor markers were also evaluated, including CA125, CA199, and CA242 at moderate and relatively high concentrations. The CR of these were all below 6.00% (Table 2), indicating that the proposed POC detection possessed excellent specificity and resisted the background of interference remarkably.

**TABLE 2:** Effect of endogenous disruptors in serum and various related tumor markers on CA50 detection.

Cross-reactant	cross-reactant concentration	Determined concentration (U mL^−1^)	Cross-reactivity (%)
Endogenous disruptors (mg ml^−1^)			
Blank control	—	29.06	—
Hemoglobin	22	27.94 ± 1.20	3.85 ± 0.43
Bilirubin	0.66	27.00 ± 0.59	−1.67 ± 0.25
Triglyceride	15	28.01 ± 0.89	−3.61 ± 0.27
Related tumor markers (U ml^−1^)			
Blank control	—	177.00	—
CA125	2,500	187.00 ± 3.96	5.72 ± 0.65
CA199	1,900	180.90 ± 2.07	1.69 ± 0.57
CA 242	500	182.56 ± 2.76	3.14 ± 0.33

The accuracy was studied through a recovery test. Different amounts of CA50 at 20.00, 140.00, and 300.00 U ml^−1^ were added to three negative human serum samples, which were analyzed five times, according to the previous method (Yang et al., 2019). The recoveries for the three different samples were 99.71%–108.37%, with all the *CVs* less than 15% (Table 3), being acceptable.

**TABLE 3:** Recovery of CA50 in the normal human serum (n = 3).

Serum sample (U ml^−1^)	Added concentration (U ml^−1^)	Determined concentration (U mL^−1^)			Recovery (%)			*CV* (%)		
		Batch 1	Batch 2	Batch 3	Batch 1	Batch 2	Batch 3	Batch 1	Batch 2	Batch 3
0.41	20.00	20.83 ± 2.28	21.56 ± 1.77	19.94 ± 0.96	104.17 ± 0.78	107.78 ± 2.01	99.71 ± 2.17	10.92	8.22	4.79
2.25	140.00	141.64 ± 1.03	147.56 ± 3.79	140.54 ± 1.10	101.17 ± 0.89	105.40 ± 0.66	100.38 ± 0.43	7.08	2.57	0.78
3.03	300.00	317.64 ± 5.58	325.10 ± 1.48	314.33 ± 4.33	105.88 ± 1.45	108.37 ± 0.99	104.78 ± 1.69	1.76	4.57	1.38

#### 3.2.4 Linearity-Dilution Effect

Here, the human serum sample containing 500 U ml^−1^ of CA50 was serially diluted with the horse serum to obtain a series of samples ranging from 0 to 500 U ml^−1^, and the calibration curve was used to check this effect. In Figure 4B, the functional equation (y = 472.02x + 6.92) was obtained by linearly fitting the concentrations of diluted CA50 and the dilution ratios. The coefficient was determined to be 0.9938, indicating that there was no effect between the human serum and the horse serum in the case of high concentrations of CA50, and the horse serum could be used to dilute the human serum samples before analysis.

#### 3.2.5 Hook Effect

The hook effect is an issue that plagues many sandwich immunoassays measuring analytes at high concentrations, which resulted in false negatives or inaccurate low value results (Fu et al., 2018). Therefore, the sample with a high-CA50 concentration was serially diluted to seek the hook effect point. From Figure 4C, the RLU linearly increased with the increasing concentration of CA50 until 1,000 U ml^−1^, while nonlinearity of the RLU became pronounced with the CA50 concentration above 1,000 U ml^−1^. These results indicated a linear relationship within the work range of 0–500 U ml^−1^. Thus, the false-negative or inaccurate low value results can be avoided in the case of samples with a high CA50 concentration.

### 3.3 Evaluation of Clinical Performance

To evaluate the clinical application feasibility of the miniaturized CLIA analyzer in this work, it was applied to analyze 114 clinical serum samples (50 negative samples and 64 positive samples) that have been determined by the large commercial CLIA analyzer (MAGLUMI^®^800) from SNIBE company. Clinical samples containing CA50 concentration over 25 U ml^−1^ were considered as positive samples (Xie et al., 2015). The results from all the 114 samples are shown in Figure 4D; the two quantitative assays showed a significant correlation (*R* ^2^ = 0.9958), indicating that the miniaturized CLIA analyzer was in good agreement with the commercial chemiluminescence analyzer. Additionally, Figure 4E showed 52 samples with concentrations of CA50 below 25 U ml^−1^ and 62 samples above 25 U ml^−1^, suggesting five false-positive samples and three false-negative samples detected by the miniaturized CLIA analyzer. Then, the concordance analysis on these samples were conducted. The positive coincidence rate and negative coincidence rate of the proposed method were 95.16 and 90.38%, respectively, and *P*_*0*_ was 92.98%. The value of Kappa also reached 0.86, being greatly higher than the acceptable value of 0.6 (Kwiecien et al., 2011). These results have demonstrated that the clinical performance of the miniaturized CLIA analyzer was highly consistent with the MAGLUMI^®^800 CLIA analyzer. Therefore, the miniaturized CLIA analyzer for detecting CA50 is promising for the application in early large-scale screening for cancer.

### 3.4 Comparison With the Existing CLIA Analyzer

The miniaturized CLIA analyzer in this study is designed for the large-scale cancer screening in resource-limited settings rather than the central clinical laboratory. The single run time of the analyzer is only 15 min to meet the requirements of rapid large-scale cancer screening. It could load 10 samples at once and realize the high throughput detection of 60 T h^−1^.

The miniaturized CLIA analyzer in this work was compared with the conventional commercial CLIA analyzer and new-type CLIA technology, respectively, in terms of assay time, detection throughput, volume, weight, and operation model (Table 4). The large conventional CLIA instruments can realize high sensitivity and throughput beyond 180 T h^−1^, however, the size of which reaches up to 2.53 m^3^ and 616 kg for Abbott i2000, as well as 0.41 m^3^ and 72 kg for the MAGLUMI^®^800 CLIA analyzer. Thus, unwieldy analyzers are not portable enough for POC testing despite the high sensitivity and throughput. The BHP9504 microtiter plate reader and Sirius2 LB 9526 tube luminometer belong to the miniaturized CLIA analyzers, possessing portable volume (0.072 m^3^; 0.034 m^3^) and weight (25 kg; 8.9 kg), respectively. But complex operation processes and the manual operation model resulted in a long assay time of 120 min (BHP9504 microtiter plate reader) and 50 min (Sirius2 LB 9526 tube luminometer), while the low sensitivity and throughput were also unsuitable for large-scale cancer screening. Moreover, the new-type CLIA was mainly aimed to develop the portable and miniaturized detection system based on microfluidics or other technologies. In Table 4, the volumes of new-type microfluidic CLIAs were all less than 0.005 m^3^ with lightweight below 10 kg. Additionally, the flow-based microarray CLIA and droplet-array microfluidic CLIA could obtain the detection results in 8 and 12 min, respectively. However, the low-throughput detection and semi-automated operating mode were their obvious deficiencies, requiring skilled professionals to perform a series of complex operations including sample pretreatment and adding samples and reagents, which is not desirable for the applications in the area with scarce medical resources (Ye et al., 2018). In contrast, the miniaturized CLIA analyzer, with an intelligent operating system developed in this study, not only had a relatively small size but also could achieve high-throughput detection without any manual operation. Therefore, shorter assay time, higher sensitivity, and more automated operation of the miniaturized CLIA analyzer for POC detection could better match the demand for the early large-scale screening of cancer.

**TABLE 4:** Comparison of the existing chemiluminescence immunoassay analyzers.

CLIA analyzer		Sample-to-answer time (min)	Detection throughput (T h^−1^)	Volume (m^3^)	Weight (kg)	Operating mode	
Commercial CLIA analyzer	Abbott i2000 (Kim et al., 2007)^a^	29	200	2.53	616	Fully automated	
	MAGLUMI^®^800 CLIA analyzer (Luang et al., 2020)^a^	17	180	0.41^j^	72	Fully automated	
	BHP9504 microtiter plate reader (Wang et al., 2007)^a^	120	<1	0.072	25	Manual	
	Sirius2 LB 9526 tube luminometer (Wang et al., 2007)^a^	50	1.2	0.034	8.9	Manual	
	**Miniaturized CLIA analyzer (this study)** ^a^	15	60	0.18	56	Fully automated	
New-type CLIA	Pump-free microfluidic CLIA (Dai et al., 2021)^b^	20^f^	3	0.0017	1.2	Semi-automated	
	Microchip-CLIA (Mou et al., 2019)^c^	60^g^	1	∼0.005^k^	NR	Semi-automated	
	Flow-based microarray CLIA (Klüpfel et al., 2021)^d^	8^h^	7.5	0.054	NR	Semi-automated	
	Droplet-array microfluidic CLIA (Huang et al., 2022)^e^	12^i^	25	0.027	10	Semi-automated	

Note: NR, not reported.

a The detected model biomarker was carbohydrate antigen 50 (CA50).

b The detected model biomarkers were alphafetoprotein (AFP), carcinoembryonic antigen (CEA), carbohydrate antigen 19–9(CA19-9), and carbohydrate antigen 125 (CA125).

c The detected model biomarkers were C-reactive protein (CRP), procalcitonin (PCT), and interleukin 6 (IL-6).

d The detected model biomarker was IgG antibodies to SARS-CoV-2.

e The detected model biomarker was procalcitonin (PCT).

f The time does not include adding samples, reagents, and substrate and washing steps.

g The time does not include adding the sample step.

h The time does not include pretreating and adding sample steps.

i The time does not include adding samples and reagents, and washing steps.

j The volume does not include the personal computer for displaying detection results.

k The volume is estimated by the reported plotting scale.

## 4 Conclusion

In this study, we developed a novel fully automated miniaturized analyzer for the detection of CA50 in the human serum. The miniaturized analyzer for CA50 detection showed a short assay time (15 min) and high-throughput performance (60 samples per hour). Batched clinical specimen analysis showed a good linear correlation between the proposed POC-CLIA and the conventional one (*R* ^2^ = 0.9958). The miniaturized analyzer is not limited to use in specialized laboratories or by skilled operators due to its compact size and simplicity in operation, thus being a powerful tool for large-scale screening of various cancers in resource-limited grassroots communities. Further work will expand the scope of assays based on the analyzer, providing a more effective solution for detecting various disease biomarkers.

## Data Availability

The original contributions presented in the study are included in the article/Supplementary Material. Further inquiries can be directed to the corresponding author.

## Abbreviations

CLIA, chemiluminescence immunoassay; CA50, carbohydrate antigen 50; CV, coefficient of variance; POC, point of care; CA199, carbohydrate antigen 199; CA1125, carbohydrate antigen 125; CA242, carbohydrate antigen 242; ALP, alkaline phosphatase; MPs, magnetic particles; SMCC, N-succinimidyl 4-(N-maleimidomethyl) cyclohexane-1-carboxylate; DTT, dithiothreitol; RLU, relative luminescence unit; LOD, limit of detection; CR, cross-reactivity; AV, average.
