# Supplementary material for: Detection of Carbohydrate Antigen 50 Based on a Novel Miniaturized Chemiluminescence Analyzer Enables Large-Scale Cancer Early Screening in Grassroots Community
Source: Front Bioeng Biotechnol. 2022 Jul 7;10:920972. doi: 10.3389/fbioe.2022.920972 (PMC9302941; doi:10.3389/fbioe.2022.920972)
Supplement: Supplementary file 1 [file Table1.DOCX]

***Supplementary material***

1. **SUPPLEMENTARY DATA**

## 1.1 EXPERIMENTAL SECTION

**1.1.1 Preparation of MPs coated** **anti-CA50 antibodies**

The previous method of coating technology was adopted (Ren et al., 2015) with a little modification. Firstly, carboxyl MPs were washed three times with 0.1 M 2-(N-morpholino) ethanesulfonic acid buffer (MES, pH 7.4). After that, carboxyl MPs were activated by directly suspending in MES buffer, containing EDC (0.2 mg/mg MPs), with gentle shaking for 15 min at room temperature. Secondly, the activation solution was discarded, and the activated MPs were washed with 2% borate buffer (pH 8.0) three times and suspended. Afterward, anti-CA50 antibodies (30 μg/mg MPs) were added into the mixed solution and incubated with MPs for 14 h at 37 ℃. Thirdly, the washing process was repeated three times with 0.05 M phosphoric buffer (containing 0.05% alkaline casein and 0.05% Tween-20, pH 7.4). Then the MPs were blocked for 1 h at 37 ℃. After the final washing step, the conjugates were resuspended in phosphate-buffered saline (pH 7.4) containing 0.3% glycine, 0.05% Tween-20 and 0.05% Proclin-300, and then stored at 4 ℃ until required, denoted as MPs-Ab.

## 1.1.2 Preparation of ALP-labeled anti-CA50 antibodies

ALP-labeled CA50 antibodies were prepared as described previously (DU Ye, 2015) with minor modifications. Firstly, appropriate SMCC was dissolved in DMSO; ALP was dissolved in 0.05 M PBS (pH 7.5). Afterward, the SMCC and ALP were mixed and incubated at room temperature for 30 min. The mixture was purified using Sephadex G-25 with PBS, with an elution velocity of 5 mL/min. Secondly, before coupling with ALP, CA50 antibodies were activated by DTT in 0.05 M PBS (pH 7.5) and then purified as the first step. After that, the activated CA50 antibodies and ALP were incubated for 1.5 h at room temperature. Next, excess ALP and antibodies in the mixture were subsequently removed using the Sephadex G-25. Finally, the purified conjugates were condensed to 1 mg/mL, added glycerol of equal volume, and then stored at 4 ℃, denoted as ALP-Ab.

## 1.1.3 The coupling efficiency of MPs-Ab

The coupling efficiency was determined by the following method. Briefly, the concentration of the mother solution of CA50 antibody (c_1_) and the supernatant of the coated MPs (c_2_) was evaluated by a Nanodrop ND-1000 spectrophotometer (LabTech, Holliston, USA). The coupling efficiency was calculated as follow:

$Coupling efficiency \left( \% \right)=(1-\frac{c_{2} \cdot v_{2}}{c_{1\cdot}v_{1}} )\times100\%$ *(1)*

Where *v*_1_ represented the addition volume of the mother solution of CA50 antibody; *v*_2_ was the supernatant volume of MPs-Ab conjugate (1).

# 1.2 RESULTS AND DISCUSSION SECTION

In this study, the ALP-AMPPD was adopted to serve as the luminous system for the fully automated POC-CLIA. The signal of ALP-AMPPD was glow type rather than flash type, stronger, more stable and long-lasting (Yu et al., 2016). These characters fulfill the clinical detection requirements. The AMPPD was catalyzed by the ALP and then produced the light signal. Undoubtedly, ALP labeling was critical for the sensitivity and accuracy of CA50 detection. Hence, we further optimized the labeling process of ALP bonded CA50 antibodies. Additionally, MPs-coating also has a remarkable influence on the luminous signal, closely linked with the sensitivity. With the optimal technology of MPs-coating, there will be a lower background and less non-specifically. A series of optimization for MPs-coating was conducted in this work simultaneously.

## 1.2.1 Selection of the optimal antibody pairs

The pair of antibodies originally affects the sensitivity and the signal of RLU for CA50 determination due to the significant difference in affinity to CA50 among different antibodies pairs. The seven combinations of anti-CA50 antibodies including Pairs 1-7 were evaluated to select the optimal antibodies for the preparation of immunoconjugates. Then, a positive sample (S7, 200 U mL^-1^) of CA50 was analyzed under all combinations. The value of RLU and the limit of detection (LOD) were calculated and compared to select the best captured and labeled antibodies for CA50 detection. The LOD was defined as the mean concentration of blank sample plus double standard deviations. As shown in Figure S1, the LOD of pair 3 (0.1099 U mL^-1^), pair 4 (0.2152 U mL^-1^), pair 5 (0.0569 U mL^-1^), and pair 6 (0.0990 U mL^-1^) performed better than that of other pairs; in addition to LOD, the highest value of RLU_S7_/RLU _S0_ was 2534 (pair 5), indicating that pair 5 possessed remarkable affinity to CA50. Therefore, to obtain higher sensitivity and RLU, C2-C1 (pair 5) was recommended.

## 1.2.2 Selection of the optimal MPs type

The types of functional groups on MPs will affect the signal of RLU and the coupling efficiency between MPs and antibodies because of the different surface characteristics and steric hindrance (Wang et al., 2007). In this study, amino-MPs, carboxyl-MPs and tosyl-MPs were evaluated. As shown in Figure S2A, the signal of RLU_S0_ of carboxyl-MPs was only 2576, significantly lower than that of amino-MPs and tosyl-MPs (*p*<0.05); in addition to RLU_S0_, a higher RLU_S7_/RLU_S0_ (818) of carboxyl-MPs was found to improve accuracy simultaneously. The highest coupling efficiency reached 87.4% with carboxyl-MPs. It was attributed to that carboxyl-MPs had less non-specific adsorption and more excellent activity to couple CA50 antibodies to enhance sensitivity and specificity. It was also notable that the RLU of tosyl-MPs was the highest among the three; however, the higher RLU_S0_ (4990) than that of carboxyl-MPs meant stronger background interference, which would decrease the sensitivity. Hence, the carboxyl-MPs were ideal solid carriers.

## 1.2.3 Optimization of the amount of EDC

The carboxyl-MPs generally need to be activated by EDC (Smith et al., 2011, Liu et al., 2014), and then the activated intermediate combines anti-CA50 antibodies efficiently. The amount of activator (EDC) has a significant effect on the capacity of MPs to bond antibodies, further affecting the coupling efficiency and RLU. Hence, EDC of different amount (0.1, 0.2, 0.35, 0.5, 0.7 mg EDC/mg MPs) was evaluated. From Figure S2B, the RLU_S0_ increased with the amount of EDC, while the RLU_S7_ increased with the increasing EDC amount and then remained stable from 0.35 to 0.7 mg EDC/mg MPs. Notably, the change in coupling efficiency was coincident with the value of RLU_S7_. These results proved that the EDC with an amount below 0.35 mg/mg MPs could not fully activate the MPs, resulting in lower signal and coupling efficiency; however, the EDC with an amount over 0.35 mg/mg MPs exceeded the demand and raised the non-specifically adsorption on MPs. The lower RLU_S7_/RLU_S0_ indicated the higher signal background with EDC amount up from 0.5 to 0.7 mg/mg MPs. A strong coupling efficiency and signal intensity with a relatively lower background were achieved when the EDC amount was 0.35 mg/mg MPs. Therefore, MPs of 0.35 mg/mg was recommended for EDC.

## 1.2.4 Optimization of the concentration of coating antibodies on MPs

The density of CA50 antibodies on MPs directly affected the capture capacity for CA50. As shown in Figure S2C, with the increasing concentration of coating antibodies, the RLU_S7_ went up until the concentration of MPs was 30 μg/mg and then kept steady. This indicated that the lack of antibodies on MPs resulted in the attenuated capture ability for CA50 when the concentration of coating antibodies changed from 10 to 20 μg/mg MPs (Chun, 2009). However, when the concentration of CA50 antibodies ranged from 40 to 50 μg/mg MPs, the RLU_S7_/RLU_S0_ kept falling, and the coupling efficiency hardly increased, suggesting that excessive CA50 antibodies on MPs did not enhance the signal but the background (Chun, 2009). At last, MPs of 30 μg/mg was selected for coating antibodies on MPs.

## 1.2.5 Optimization of the coupling time

The coupling time would directly affect the coupling efficiency between antibodies and MPs (Chun, 2009). The MPs were not coated with enough antibodies to capture CA50 because of too short coupling time. In Figure S2D, the RLU and coupling efficiency both increased with the increasing coupling time and reached the peak at the 14th hour. There was no significant difference in coupling efficiency and RLU at the 14th and 16th hours, indicating that 14 hours were enough for the MPs to combine sufficient coating antibodies.

## 1.2.6 Selection of optimal cross-linker for ALP labeling CA50 antibodies

The combination of antibodies with the ALP relied on different types of cross-linkers. In this study, glutaraldehyde, sodium periodate, and SMCC were evaluated by the RLU. As shown in Figure S3A, different cross-linkers exerted a significant influence on the RLU. Among the cross-linkers, a relatively low RLU was observed with the cross-linkers of glutaraldehyde. In contrast, the highest RLU (S_7_) was obtained using sodium periodate to bond the ALP and antibodies, indicating the relatively high labeling efficiency; but the relatively high RLU_S0_ (2563) of sodium periodate caused strong background. It was worth noting that the highest value of RLU_S7_/RLU_S0_ (2461) was found when SMCC served as the cross-linkers. This showed the relatively low background and strong RLU of the positive sample when SMCC acted as the cross-linkers, indicating that SMCC processed excellent stability and activity because of its special spatial structure. Therefore, SMCC applied to link ALP with antibodies could obtain a continuously stable signal.

## 1.2.7 Optimization of SMCC concentration

The concentration of SMCC could impact the labeling efficiency; thus, it was investigated at the range of 2 to 15 mg mL^-1^in this study. In Figure S3B, the RLUs increased with the increasing concentration of SMCC (0-10 mg mL^-1^) and remained unchanged when the concentration was 10-15 mg mL^-1^. It indicated that the concentration of 10 mg mL^-1^ was saturated for SMCC. Simultaneously, the highest value of RLU_S7_/RLU_S0_ (1921) was obtained at the concentration of 10 mg mL^-1^. Therefore, the optimal concentration of SMCC was 10 mg mL^-1^.

## 1.2.8 Stability analysis of the immunoreagents

The stability of immunoreagents played a critical role in immunoassay and would affect the precision and sensitivity of assay. The immunoreagents generally were stored at 2-8 ℃ (Khramtsov et al., 2019). According to the previous study (Crowther, 2000), immunoreagents at 37 ℃ for 7 days are equivalent to those at 4 ℃ for 12 months. Hence, the MPs-Ab and ALP-Ab were stored at 37 ℃ for 7 days. Then the sensitivity, precision, and correlation coefficient (*R*) of the calibration curve were synthetically evaluated. Table S1 showed that those values had some slight fluctuation after 7 days at 37 ℃ but still met the standard of clinical demand. These results also demonstrated that the binding of MPs-Ab and ALP-Ab complexes was tight; the technical optimization of MPs coating and ALP labeling enabled the LOD, precision and *R* of the calibration curve to remain stable at 37 ℃ for 7 days.

**2 SUPPLEMENTARY TABLES AND FIGURES**

**2.1 SUPPLEMENTARY FIGURES**

**Figure captains**

Figure S1 Selection of the optimal antibody pairs. A1 labeling- A2 coating (pair 1), A2 labeling with A1 coating (pair 2), B1 labeling with B2 coating (pair 3), B2 labeling with B1 coating (pair 4), C2 labeling with C1 coating (pair 5), D1 labeling with D2 coating (pair 6), and D2 labeling with D1 coating (pair 7).

Figure S2 Optimization of the technology of MPs coating. (A) optimizing the type of MPs; (B) optimizing the amount of EDC (activator); (C) optimizing the concentration of CA50 antibody on MPs; (D) optimizing the coupling time of MPs and CA50 antibodies.

Figure S3 Optimization of the technology of ALP labeling. (A) optimizing the type of cross-linker; (B) optimizing the concentration of SMCC (cross-linker).


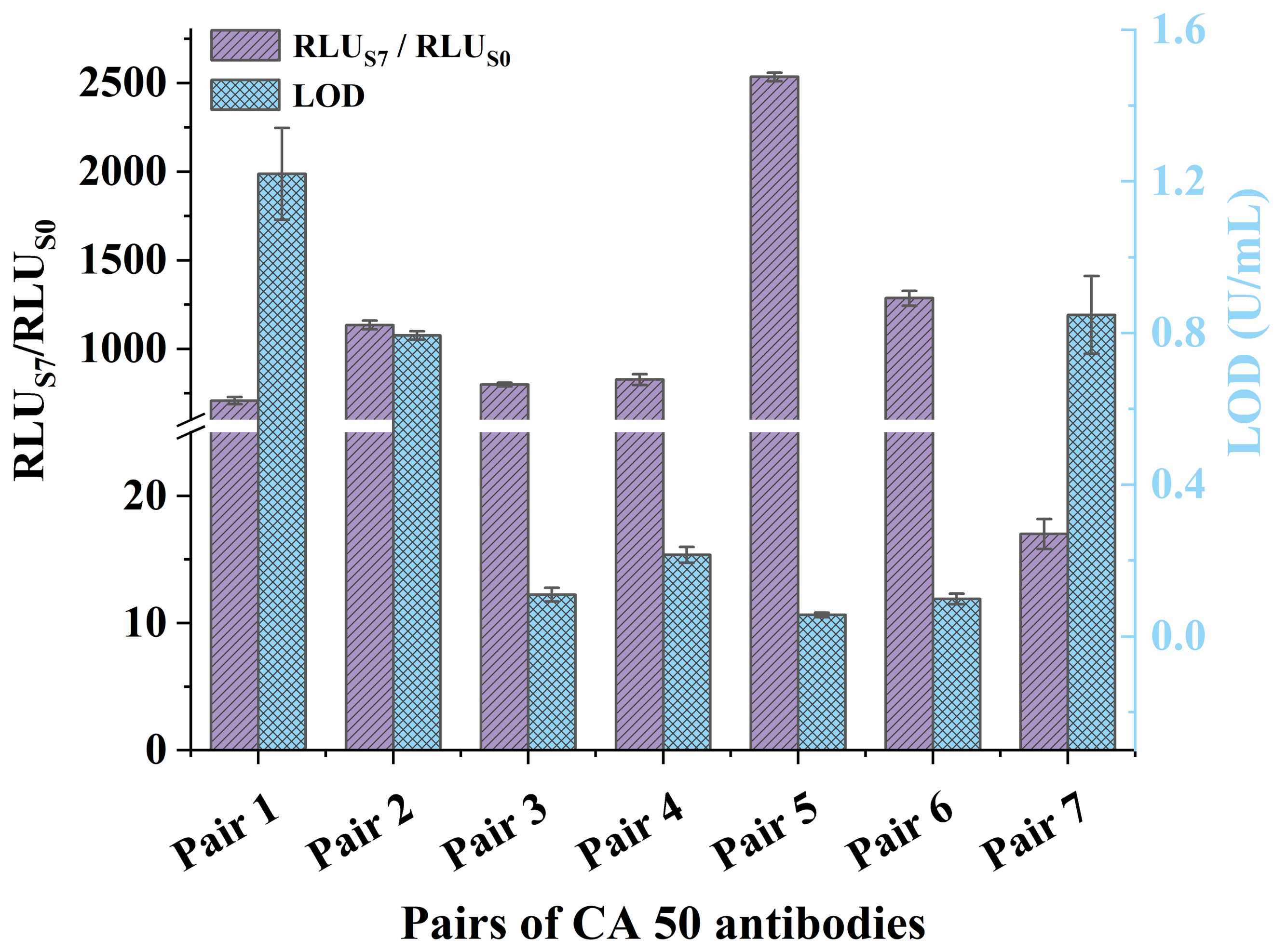


**Figure S1**

**

**

**Figure S2**

**

**

**Figure S3**

**2.2 SUPPLEMENTARY TABLES**

**Table S1 Evaluation of the** **accelerated stability of immunoreagents for three batches.**

| **Batch** | **The correlation coefficient of the calibration curve (*R*)** | | **LOD (U mL^-1^)** | | ***CV* of Precision (%)** | |
| --- | --- | --- | --- | --- | --- | --- |
|  | 4 ℃/7d | 37 ℃ / 7d | 4 ℃/7d | 37 ℃ / 7d | 4 ℃/7d | 37 ℃ / 7d |
| Batch 1 | 0.9935 | 0.9902 | 0.057 | 0.045 | 4.05 | 6.56 |
| Batch 2 | 0.9956 | 0.9966 | 0.061 | 0.069 | 5.32 | 8.18 |
| Batch 3 | 0.9990 | 0.9941 | 0.053 | 0.065 | 7.63 | 8.79 |
| Acceptance criteria | ≥ 0.9800 | | ≤ 1.00 | | ≤ 15.00 | |
| Whether or not meet the criteria | Yes | | Yes | | Yes | |

**Reference**

CHUN, L. J.-T. L. R. P. W. M. X. L. (2009). Detection of Human Chorionic Gonadotropin by Highly Sensitive Magnetic Enzyme-linked Chemiluminescent Immunoassay. *Chinese Journal of Analytical Chemistry,* 37**,** 985-988.

CROWTHER, J. R. (2000). The ELISA guidebook. *Methods Mol Biol,* 149**,** Iii-iv, 1-413. doi: 10.1385/1592590497.

DU YE, L. J., PENG BO (2015). Process comparison of conjugating alkaline phosphatase with anti-human chorionic gonadotropin antibody. *Mol Diagn Ther,* 7**,** 333-340.

KHRAMTSOV, P., BARKINA, I., KROPANEVA, M., BOCHKOVA, M., TIMGANOVA, V. & NECHAEV, A. (2019). Magnetic Nanoclusters Coated with Albumin, Casein, and Gelatin: Size Tuning, Relaxivity, Stability, Protein Corona, and Application in Nuclear Magnetic Resonance Immunoassay. *Nanomaterials (Basel),* 9, 1345. doi: 10.3390/nano9091345.

LIU, J., ZHANG, L., WANG, Y., ZHENG, Y. & SUN, S. (2014). An improved portable biosensing system based on enzymatic chemiluminescence and magnetic immunoassay for biological compound detection. *Measurement,* 47**,** 200-206. doi: 10.1016/j.measurement.2013.08.057.

REN, Z. Q., LIU, T. C., ZHUANG, S. H., LIN, G. F., HOU, J. Y. & WU, Y. S. (2015). Establishment of Magnetic Microparticles-Assisted Time-Resolved Fluoroimmunoassay for Determinating Biomarker Models in Human Serum. *PLoS One,* 10, e0130481. doi: 10.1371/journal.pone.0130481.

SMITH, J. E., SAPSFORD, K. E., TAN, W. & LIGLER, F. S. (2011). Optimization of antibody-conjugated magnetic nanoparticles for target preconcentration and immunoassays. *Anal Biochem,* 410**,** 124-32. doi: 10.1016/j.ab.2010.11.005.

WANG, X., LIN, J. M. & YING, X. (2007). Evaluation of carbohydrate antigen 50 in human serum using magnetic particle-based chemiluminescence enzyme immunoassay. *Anal Chim Acta,* 598**,** 261-7. doi: 10.1016/j.aca.2007.07.052.

YU, S., YU, F., LIU, L., ZHANG, H., ZHANG, Z., QU, L. & WU, Y. (2016). Which one of the two common reporter systems is more suitable for chemiluminescent enzyme immunoassay: alkaline phosphatase or horseradish peroxidase? *Luminescence,* 31**,** 888-92. doi:10.1002/bio.3047.
